# The last generation of the pommy medical officer

**DOI:** 10.1007/s40037-013-0048-1

**Published:** 2013-03-15

**Authors:** A. Armstrong

**Affiliations:** 1 Princess Elizabeth Orthopaedic Centre, Royal Devon and Exeter Hospital, Gladstone Road, Exeter, Devon UK; 2 44a Fore Street, Topsham, Exeter, EX3 0HY Devon UK

In recent years, there has been an influx of British doctors to Australia every August, each with a different personal agenda. Some look to widen their clinical and educational experience before embarking on specialist training. Others seek respite care from the National Health Service, whilst many are looking for a lifestyle change and choose to stay. Whatever the motivation, this opportunity for Poms may be on the wane as the Australian trained doctors fill the vacancies.

Consequently, I might be the remnant of a dying breed. This is why I am counting my blessings.

Last year had a harsh winter in the ‘outback’ that is the Quantock Hills of Somerset, in the UK. The relentless falling of snow resulted in Musgrove Park Hospital, a large district general hospital, working on skeleton staff over a busy time of year. Luckily, I lived over the road and was just about able to make the short commute in my wellington boots and duffel coat to boost the small existing workforce. I was covering three busy orthopaedic wards and doing the on-call shift too because we were particularly light staffed that day. In the true old-school English fashion with a stiff upper lip I was told by my consultant: ‘You’ll cope Armstrong!’

I was in my Foundation Year 2, a critical time in my career with job applications around the corner. I was unsure what I wanted to do: apply for specialist training, do a Master’s or maybe perhaps go to Australia? As I gazed out onto the six-foot snow drifts with my pager relentlessly going off, Australia seemed a very attractive option.

The Managing Medical Careers (MMC) movement in 2003 reformed the UK system by streamlining junior training. Many felt this was a restrictive ‘conveyor belt’ with little flexibility. The opportunity to discover subspecialities, which was previously encouraged in the Senior House Officer years, had been lost. Moreover, applying for registrar training had become unfeasible for many junior trainees with competition ratios as high as 15 to 1 in some surgical specialities. Those doctors who were unsuccessful at national selection were forced to build their own surgical experience outside of official training and were thus named ‘The Lost Tribe’ [1].

Professor Jonathan Tooke led an independent inquiry into the MMC in 2009 and criticized its efficacy. Tooke stated that training roles were unclear and that there was a lack of cohesion between education and service provision. The report suggested further restructuring of postgraduate medical education making training more flexible for junior trainees [2]. Despite the report being well received amongst the medical profession only a quarter of the suggestions were accepted by the Department of Health.

The lack of training experience was compounded further by the introduction of the European Working Time Directive, limiting trainees to 48 h of work per week. Many consultants felt the trainees were not getting enough experience and this was most pronounced in surgery with reduced time in the operating theatre.

Like many British doctors I wanted to gain further experience prior to applying to training in the UK. My clinical experience up to this time had not prepared me sufficiently for the very competitive process of applying for the speciality of surgery. A number of Australian hospitals offer a Senior Resident Medical Officer role and the chance to rotate through different specialities; this seemed ideal for me.

I was fortunate to spend a year at The Prince of Wales Hospital, Sydney, New South Wales. Here, I was offered a surgical rotation in orthopaedics, neurosurgery and cardiothoracic surgery—my areas of professional interest. I enjoyed focussed training and my clinical supervisors took a special interest in my development. I assisted in some complex, groundbreaking neurosurgery. These experiences have been invaluable and in my circumstance would have been difficult to replicate in the UK system.

My Australian work experience proved crucial in my application for surgical training in the UK, when I was able to secure my first choice training programme, in my preferred area of England.

Times are rapidly changing as the Australian government is striving for a more self-sufficient system, not relying so heavily on foreign graduate doctors. There are more sponsored places at medical school and as a result jobs previously filled by foreign graduates are fewer and farther between.

A recruitment campaign started in 2010 with the recognition that the healthcare system required a number of doctors that exceeded those being trained in Australia. This shortfall was bridged by the employment of overseas trained practitioners. The Clinical Education and Training Institute of New South Wales produced an analytical paper of the existing workforce in 2010. This paper concluded to provide funding for an increase in intern positions from 670 (in 2010) to 1,050 by 2015 [3]. Thus there was also a knock-on requirement of funding for new positions in specialist training.

Whatever the motivation, one opinion was unanimous amongst British doctors: practising in Australia is an invaluable method of securing wider professional experience. I am grateful for the opportunity that Australia has offered but I suspect similar opportunities will not be as readily available in the future.
